# *Enterobacteriaceae* dominate the core microbiome and contribute to the resistome of arugula (*Eruca sativa* Mill.)

**DOI:** 10.1186/s40168-019-0624-7

**Published:** 2019-01-29

**Authors:** Tomislav Cernava, Armin Erlacher, Jung Soh, Christoph W. Sensen, Martin Grube, Gabriele Berg

**Affiliations:** 1 Institute of Environmental Biotechnology, Graz University of Technology, Petersgasse 12, 8010 Graz, Austria; 2 Institute of Computational Biotechnology, Graz University of Technology, Petersgasse 14, 8010 Graz, Austria; 3 Institute of Plant Sciences, University of Graz, Holteigasse 6, 8010 Graz, Austria; 4 BioTechMed Graz, Mozartgasse 12/II, 8010 Graz, Austria

## Abstract

**Background:**

Arugula is a traditional medicinal plant and popular leafy green today. It is mainly consumed raw in the Western cuisine and known to contain various bioactive secondary metabolites. However, arugula has been also associated with high-profile outbreaks causing severe food-borne human diseases. A multiphasic approach integrating data from metagenomics, amplicon sequencing, and arugula-derived bacterial cultures was employed to understand the specificity of the indigenous microbiome and resistome of the edible plant parts.

**Results:**

Our results indicate that arugula is colonized by a diverse, plant habitat-specific microbiota. The indigenous phyllosphere bacterial community was shown to be dominated by *Enterobacteriaceae,* which are well-equipped with various antibiotic resistances. Unexpectedly, the prevalence of specific resistance mechanisms targeting therapeutic antibiotics (fluoroquinolone, chloramphenicol, phenicol, macrolide, aminocoumarin) was only surpassed by efflux pump assignments.

**Conclusions:**

Enterobacteria, being core microbiome members of arugula, have a substantial implication in the overall resistome. Detailed insights into the natural occurrence of antibiotic resistances in arugula-associated microorganisms showed that the plant is a hotspot for distinctive defense mechanisms. The specific functioning of microorganisms in this unusual ecosystem provides a unique model to study antibiotic resistances in an ecological context.

**Electronic supplementary material:**

The online version of this article (10.1186/s40168-019-0624-7) contains supplementary material, which is available to authorized users.

## Background

Plants are generally colonized by a vastly diverse microbiota, which contributes to the host plant’s health and productivity [40, 46]. However, the structure, abundance, and occurrence of microorganisms can be variable across different plant habitats [51, 62] and plant species/genotypes [9, 15]. All members of the *Brassicaceae* plant family characterized thus far have shown an extraordinary degree of specificity in their associated microbiota [6, 7, 65]. They are known for a bacteria-dominated composition of the microbiome, as they do not harbor a typical mycorrhiza [30, 54]. The effective defense mechanisms of the plant are based on a unique composition of antimicrobial secondary metabolites, including glucosinolates [42, 52]. Their resulting degradation products (isothiocyanates) have also received scientific interest, as they might be involved in cancer prevention [31]. Arugula (*Eruca sativa* Mill.) is the only salad plant from this family, which is quite popular for human consumption. Commonly known as rucola (or garden rocket, respectively), it has its origin in the Mediterranean and has been cultivated at least since Roman times [43, 68]. The unique aroma is caused mainly by the aforementioned isothiocyanates and butane derivates [37], respectively. In addition, arugula also contains a number of other health-promoting agents [5]. While the health-promoting properties of arugula are undisputed [2], almost nothing is known about the associated microbiota. Our hypothesis was that arugula would be associated with a highly specialized microbiota, due to metabolites it produces, which were expected to be highly different from salad species from the *Asteraceae* family, which have been well studied previously [18, 53].

Arugula has been reported to be the potential source of pathogen outbreaks, especially associated with *Enterobacteriaceae* [32, 50]. Only in recent years, *Enterobacteriaceae* have been confirmed as indigenous components of the plant microbiome in other species [12, 26, 27, 60]. These pathogenic bacteria come also into focus for their potential role in the transfer of antibiotic resistance by mobile genetic elements [28, 34, 58]. The environmental resistome has now been recognized as the origin and reservoir of antibiotic resistance genes and is considered to be dynamic and ever-expanding [11]. With increasing resistances, the success of treatment options for infectious diseases, e.g., cases of sepsis, after major surgery or during cancer chemotherapy will be compromised [20, 22]. Wellington et al. [66] discussed an extensive antibiotic resistance (AR) “pollution” in our environment. In a large-scale metagenomics-based study of antibiotic resistances in the environment, Nesme et al. [49] depicted soil habitats as the most diverse reservoir for AR across four different environmental clusters. Lettuce was also reported as a source of multi-resistant bacteria [17, 64], but such information thus far lacks for arugula. Therefore, we were interested in the potential for antibiotic resistance in this unique ecosystem and hypothesized that the arugula resistome may be affected by its particular microbiome.

We have investigated the arugula phyllosphere in comparison with the rhizosphere and the surrounding bulk soil, using whole metagenome sequencing and functional annotation of the resulting genomic sequences. In addition, complementary amplicon sequencing to further characterize the *Enterobacteriaceae* populations was performed in order to complete the integrative study of the arugula microbiome.

## Materials and methods

### Sampling of arugula plants

Arugula plants were grown in garden soil (hereafter referred to as bulk soil) in a suburban region of Graz (Austria; approx. 47°4′ 13′′ N 15° 28′ 19″ E). All samples used are summarized in Additional file 1: Table S1. Edible plant parts consist of elongated leafs with short stalks and are termed as phyllosphere throughout this study when referring to the microbial habitat. The microbial fraction of the *Eruca sativa* system was extracted separately for both the phyllo- and rhizosphere in two consecutive samplings. During the first sampling, arugula plants were collected to assess the enterobacterial microbiome by amplicon sequencing of 16S rRNA gene fragments. Plants were harvested in their final growth stage of leaf development in July. During the follow-up sampling, samples from the same garden were obtained in early November at their final growth stage when the plants had already formed flowers and seed bodies. They were grown in a warm summer with continental or hemiboreal climate, and the recorded climate profiles (Additional file 1: Figure S1), which started 2 weeks before sampling, show relatively warm temperatures, moderate but fluctuating precipitation, sunshine and atmospheric pressure levels, overall low wind levels, and average air humidity. In addition to the phyllo- and rhizosphere samples, bulk soil was sampled for metagenome sequencing. All samples were stored on ice and immediately processed after arrival at a nearby laboratory.

### Amplicon sequencing and processing of enterobacterial 16S rRNA gene fragments

#### Sample preparation

The microbial fractions were extracted separately for the rhizo- and phyllospere, and three independent single replicates per habitat, consisting of 15–20 leaves or roots, respectively, were collected and stored separately. For the homogenization of the samples, 5 g of plant material per replicate was physically disrupted with a sterile pestle and mortar, re-suspended in 10 ml of 0.85% NaCl, transferred in two 2 ml Eppendorf tubes, and subsequently centrifuged (16,500 g, 20 min, 4 °C). The pellet obtained was used for isolation of the total-community DNA with the FastDNA® SPIN Kit for Soil (MP Biomedicals, Solon, OH, USA). For mechanical lysis, the cells were homogenized twice in a FastPrep® FP120 Instrument (Qbiogene, BIO101, Carlsbad, CA, USA) for 30 s at a speed of 5.0 m s^−1^ and treated according to the manufacturer’s protocol.

#### Amplification and sequencing of 16S rRNA fragments

The 16S rRNA gene fragments of the *Enterobacteriaceae* were amplified in a dual-phase nested PCR approach using multiplex identifier (MID) tagged primers. The PCR was conducted with specific primers for enterics, using Entero-F234 and Entero-R1423 (V3-V8), according to the method described by Binh et al. [10]. The nested PCR was carried out as described by Heuer et al. [33] using the primer pair F984 and R1378 modified for multiplex 454 sequencing according to the specification. The products of two independent PCR reactions per sample were pooled and purified using the Wizard SV Gel and PCR Clean-Up System (Promega, Madison, USA). Purified PCR products were pooled (200 ng each) and sequenced on a Roche GS FLX+ 454 Titanium platform (Macrogen Korea, Seoul, South Korea).

#### Bioinformatics sequence processing

Sequences were analyzed with the QIIME software version 1.8.0 [16]. MID, primer, and adapter sequences were removed, and the sequences were quality- (minimal score 50) and length filtered (minimal raw fragment length: E1 ≥ 350; E2 and E3 ≥ 430), followed by a denoising step (using the denoise wrapper and denoiser script; both contained within the QIIME pipeline). Chimeras and remaining sequences of non-target, plastidal, and mitochondrial origin were removed. OTU tables were created with UCLUST [25] at a 100% cut-off level. Unique reads were classified with the RDP classifier [63] v2.5 and the NCBI RefSeq database. The datasets were rarified on a naїve Bayesian classifier [63] to the number of least reads within each experiment, to compute alpha and beta diversity indices. An OTU-based network correlating the OTUs at 100% cut-off level was constructed with Cytoscape v.3.1.0 [57]. Centiscape v.2.2 [55] was used to calculate centroid values for each node of the network and label them accordingly.

### Metagenome shotgun sequencing and bioinformatic processing

#### Sample preparation

Five grams of each sample (phyllo-, rhizosphere, and bulk soil) were weighed, transferred into sterile plastic bags together with 10 ml 0.85% NaCl, and mechanically processed twice for 210 s in a Bagmixer (Interscience, St. Nom, France). Samples were placed at interims of 5 min on ice. Homogenized cell suspensions were further transferred into S34 tubes, and centrifuged at 10,000 rpm for 20 min. The supernatant was discarded for each approach and the cell pellets were stored at − 20 °C. In total, we processed 24 × S34 tubes (35 ml/S34) foliage samples from a total 90 bags (cell pellets were stacked three times and the supernatant discarded between each centrifugation round in order to increase the total yield), 8 × S34 tubes (35 ml/S34) rhizosphere samples from a total of 35 bags, and 6 × S34 tubes (35 ml/S34) soil samples from a total of 35 bags, respectively. DNA was extracted using the FastDNA® SPIN Kit for Soil (MP Biomedicals, Solon, OH, USA) from six pellets per habitat (foliage, rhizosphere, soil) using 300 mg each of the crude cell pellets and processing as stated in the manufacturer’s protocol. The DNA samples were eluted in 100 μL H_2_O_ultra pure_ and checked for quality and quantity using NanoDrop Photometer. Then, 2 μg of each replicate were pooled for a total 12 μg of metagenomic DNA per habitat.

#### Shotgun sequencing of total community DNA

Metagenomic sequencing was performed on an Illumina HiSeq2000 system (2 × 150 bp) by Eurofins MWG Operon (Ebersberg, Germany) following the Eurofins MWG Operon protocol.

#### Sequence quality filtering

The raw read data were uploaded to the Galaxy Main server (http://usegalaxy.org) and processed using the FASTQ Joiner, FASTQ Groomer, FASTQ Quality Trimmer, and Filter FASTQ (min. length = 75, min. quality = 20, allowed outside quality range = 2). For each of the datasets, only high-quality reads were retained for further processing, resulting in the following read numbers to process further: phyllosphere: 41,867,724 (79.8%), rhizosphere: 35,463,395 (74.7%), and soil: 27,085,866 (79.3%).

#### Reference alignment

Reads were aligned to the reference genomes of *Brassica oleracea* (BOL; ncbi.nlm.nih.gov/genome), *Brassica rapa* (Brapa_1.0), and *Raphanus sativus* (Rs1.0), respectively, with the goal of discarding aligned reads in order to reduce plant-derived genetic bias. To align the quality-filtered reads to the reference genomes, Bowtie 2 [39] was used, with the default alignment parameters. Additional file 1: Table S2 shows the alignment results. Those reads that aligned to at least one of the three genomes were excluded from further processing. Thus, only those reads that were not aligned to any of the three reference genomes were used as input to the assembly program. The phyllosphere and soil samples had the largest and smallest numbers of aligned reads among the three samples, respectively, which was to be expected.

#### Sequence assembly

Assembly of the reads into contigs was performed using the Velvet (version 1.2.10; [67]) de novo assembly software. Since the choice of the *k*-mer length is crucial, multiple assemblies with different *k* values were performed on each sample in order to determine the best *k*-mer length. In all assemblies, the expected coverage value was automatically found by Velvet and the insert length for paired-end reads was set at 350. Additional file 1: Table S3 shows the key statistics of the final assemblies, where *k* is the length of the *k*-mer used. Only those contigs that had at least 90% of the median coverage and were at least as long as twice the *k* value were selected for subsequent analysis.

#### Taxonomic and functional analyses

For each sample, taxonomy and functional analyses were performed using the MEGAN program (version 5.11.3; [36]). For taxonomic analyses, the blastn program was run on all the filtered contigs on the TimeLogic DeCypher boards (www.timelogic.com), against the “nt” database with 34,646,553 nucleotide sequences downloaded from NCBI on February 12, 2016. In order to create input for functional analyses, the blastx (nucleotide-to-protein) program was run on all the filtered contigs on the TimeLogic DeCypher boards, against the “nr” database with 81,622,391 protein sequences downloaded from NCBI on February 12, 2016. The blastn results from each sample were imported into MEGAN to perform the taxonomy analysis for the sample, where each contig is assigned to a bacterial lineage at the lowest rank possible with sufficient confidence. An identical number of contigs assigned to bacteria (the smallest such number among the phyllosphere, rhizosphere and bulk soil samples) were randomly extracted from each sample and used to compare subsequent bacterial taxonomic and functional analyses results among the three samples. Therefore, the taxonomy and functional analyses of the bacteria are based on the blastn and blastx results, respectively, for the 89,462 subsampled contigs from each sample. Similarly, the taxonomy and functional analyses of *Enterobacteriaceae* are based on the blastn and blastx results, respectively, for the 5494 subsampled contigs from the phyllosphere and rhizosphere samples, respectively. A very low number (356) of contigs from the soil sample was assigned to the *Enterobacteriaceae*; therefore, we were not able to include them in a meaningful comparison.

#### Antibiotic resistance analysis

Microbial antibiotic resistance profiles were analyzed using the Comprehensive Antibiotic Resistance Database (CARD; [45]). The antibiotic resistance protein sequences for metagenomic data were downloaded from the CARD website (card.mcmaster.ca) as a FASTA file and used as the target sequences for the nucleotide-to-protein Blast (blastx) analysis of the assembled contigs for each sample. The analysis was performed by using only the top blast hits, with a percentage identity of at least 50%. A single hit of a contig to an antibiotic resistance protein was counted as multiple antibiotic resistance category hits, if the protein mapped to multiple categories.

#### MG-RAST analysis of non-assembled reads

Abundances within the enterobacterial fraction were analyzed in a separate bioinformatics approach based on non-assembled metagenome sequences. The metagenomes (phyllosphere, rhizosphere, and bulk soil) were uploaded on the Metagenomic RAST (MG-RAST) server [47] and initially processed using the default parameters. The processing included the removal of artificial replicate sequences [29], low-quality sequences [21], short sequences, and sequences containing ambiguous bases. Phylogenetic annotations were obtained from the RefSeq database, with a maximum *e* value of 10^−5^, minimum identity of 60%, and minimum alignment length of 15 bp for RNA databases.

### Isolation of a strain collection and antibiotic susceptibility tests

For the isolation of enterobacteria, homogenized cell suspensions from the second sampling were plated on growth media. In total, 180 aerobic bacteria were isolated from the arugula phyllosphere and rhizosphere as well as bulk soil. Different media including PDA, MacConkey, KingsB, R2A (all obtained from Carl Roth GmbH, Karlsruhe, Germany), MIS (prepared according to [61]), and NAII (Sifin, Berlin, Germany) allowed a broad-spectrum coverage to create a representative strain library. None of the media used for the initial isolation was supplemented with antibiotics, in order to avoid an isolation bias caused by selective conditions. A disk diffusion antimicrobial susceptibility test method [35] was used to test for susceptibility towards a defined set of antibiotics. Instead of MH agar, NAII agar was used for the test plates. Unique morphotypes were tested for their sensitivity against Ampicillin (10 μg), Chloramphenicol (30 μg), Erythromycin (15 μg), Gentamicin (10 μg), Penicillin G (10 Units; 6 μg), Polymyxin B (300 Units; 30 μg), Streptomycin (10 μg), and Tetracycline (30 μg).

## Results

### The arugula microbiome and the community structure of the enterobacterial fraction

**Fig. 1:**
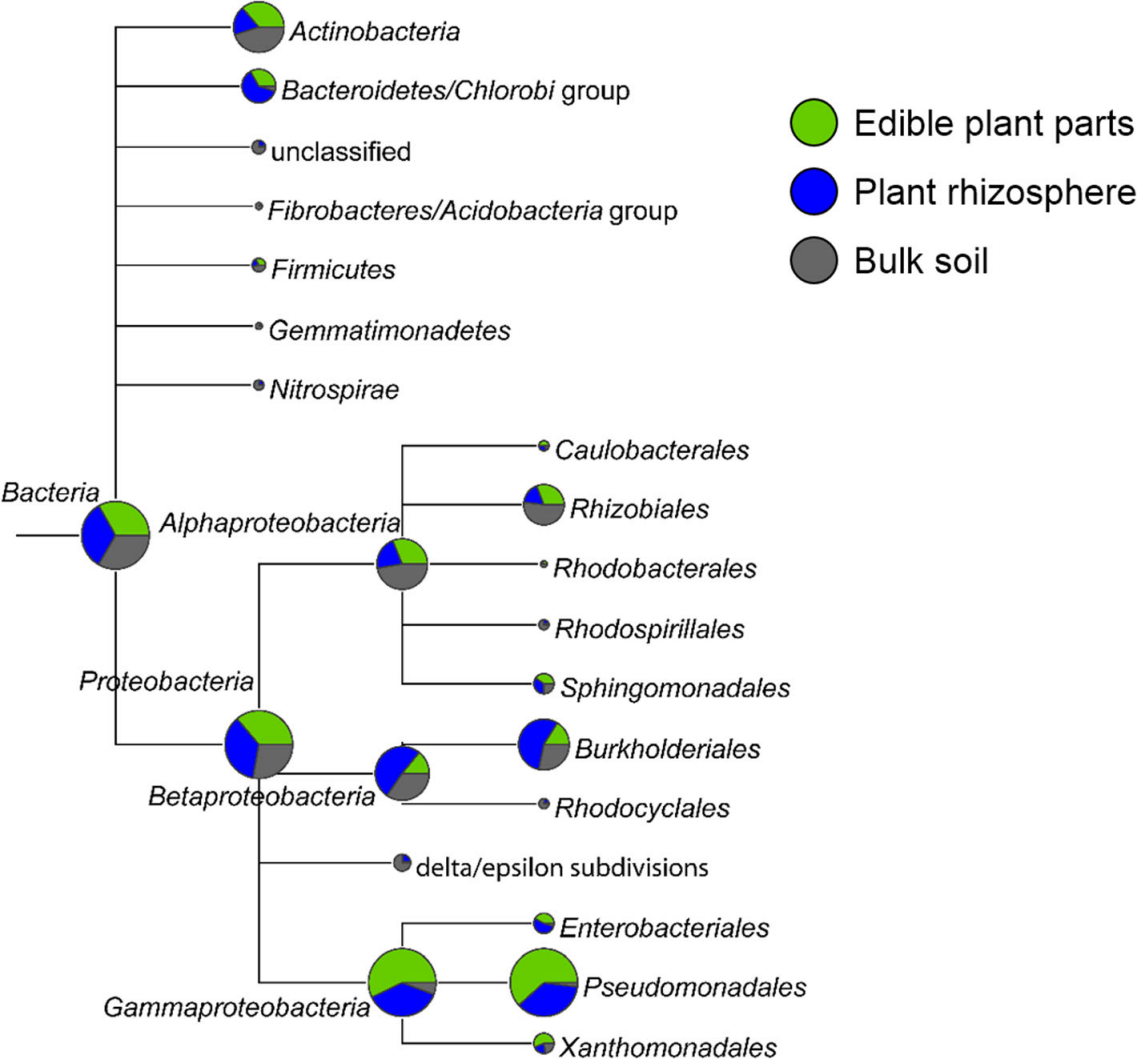
Composition of the bacterial biota in the three analyzed samples. Circles represent the square root scaled taxonomic structure of the assembled metagenomes of the phyllosphere (green), rhizosphere (blue) and bulk soil (gray). Only abundant taxa (*n* > 1000 hits) were plotted using MEGAN (v.5.7)

Assembly-based community structure analyses of habitat-specific metagenomes showed a high proportion of contigs assigned to *Proteobacteria*, which were relatively evenly distributed among the analyzed habitats (phyllosphere 66,829; rhizosphere 66,108; soil 50,993; Fig. 1). Within this predominant phylum*,* the bacterial class *Gammaproteobacteria* was most abundant and almost exclusively mapped with contigs derived from plant associated habitats (p: 46,726, r: 30,379, s: 4626). Within this class, the order *Pseudomonadales* (p: 39,767, r: 23,446, s: 1280) accounted for most contigs, but *Enterobacteriales* (p: 3225, r: 4051, s: 356) and *Xanthomonadales* (p: 3341, r: 1279, s: 1322) were also abundant. Other taxa that do not belong to *Gammaproteobacteria* but were highly abundant, especially in the rhizosphere, were *Bacteriodetes* (p: 5629, r: 10,469, s: 831) and *Burkholderiales* (*Betaproteobacteria*; p: 5970, r: 20,666, s: 10,495). *Actinobacteria* (p: 14,382, r: 7424, s: 17,894) and *Rhizobiales* (*Alphaproteobacteria*; p: 7362, r: 4101, s: 12,368) were predominant in the bulk soil metagenome.

**Fig. 2:**
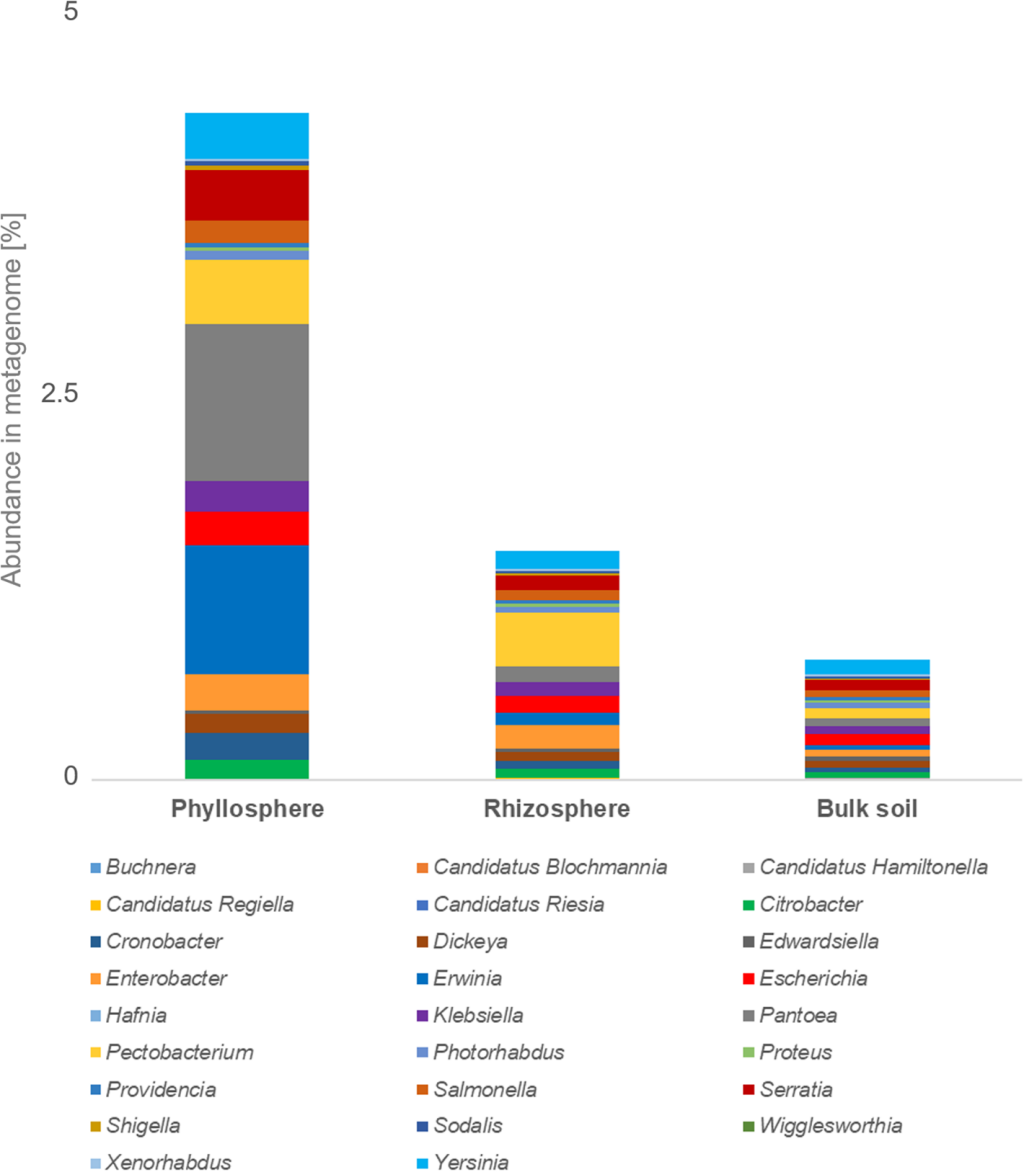
Structure and abundance of enterobacterial population in the metagenomes. Only those taxa with an assigned read number higher than 100 in at least one metagenome are shown. The chart illustrates the distribution and abundance of identified lineages of *Enterobacteriaceae*. Relative abundances within the bacterial fraction are based on non-assembled reads with taxonomic assignments in NCBI’s RefSeq database (ncbi.nlm.nih.gov/refseq)

In order to further characterize the enterobacterial community, an MG-RAST comparison was implemented (Fig. 2) focusing on the important bacterial family of *Enterobacteriaceae*. This assessment is based on non-assembled reads and thus provides a higher number of hits, when compared to the contig-based analyses. Alignments showed that the habitats harbored a similar enterobacterial diversity; however, there were substantial variations in abundances between the metagenomes. Remarkably, a nearly threefold higher abundance of *Enterobacteriaceae* was observed in the phyllosphere, when compared to the rhizosphere (relative abundance in p: 4.3%, r: 1.5%, s: 0.8%). The most abundant genera therein were *Pantoea* (p: 1%, r: 0.1%, s: 0.1%) and *Erwinia* (p: 0.8%, r: 0.1%, s: < 0.1%), followed by *Pectobacterium* (p: 0.4%, r: 0.4%, s: 0.1%), *Serratia* (p: 0.3%, r: 0.1%, s: 0.1%), and *Yersinia* (p: 0.3%, r: 0.1%, s: 0.1%). *Pectobacterium* was the only prevailing genus that was found in similar abundance in the phyllosphere and rhizosphere metagenomes and at the same time the most abundant genus in the rhizosphere.

### The enterobacterial core community

**Fig. 3:**
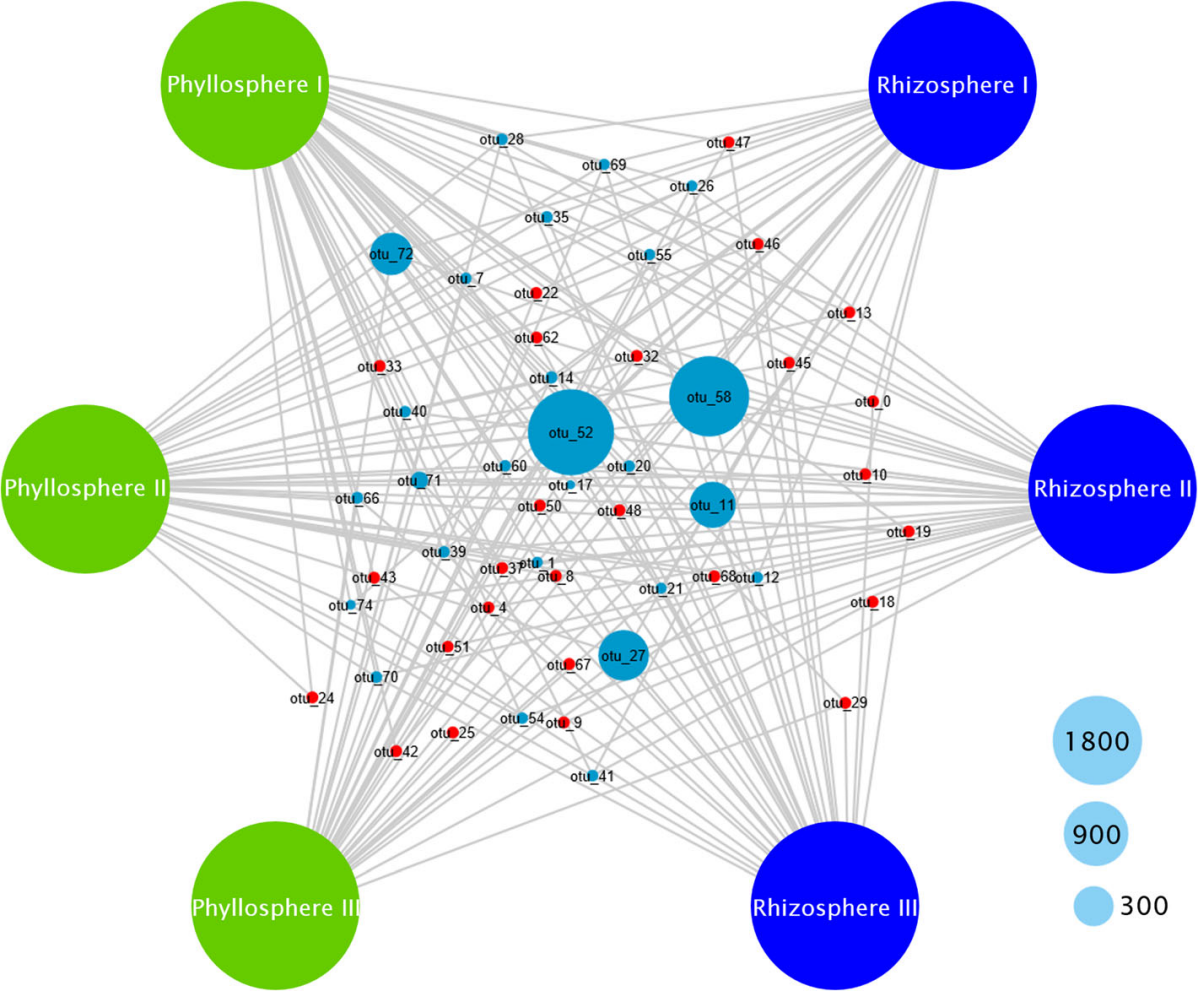
Enterobacterial core microbiome in the arugula phyllo- and rhizosphere. The OTU-based network correlates OTUs at a 100% cut-off level. Cytoscape v.3.1.0 was used for network rendering and Centiscape v.2.2 to calculate centroid values. OTUs that are above the defined centrality threshold are labeled in blue. The node size correlates with the number of assigned reads; three reference node sizes are visualized in the legend. Taxonomic assignments of the predominant OTUs are provided in Additional file 1: Table S4

The analysis of 16S rRNA gene fragment amplicons provided deeper insight into the indigenous enterobacterial microbiome at OTU-based taxonomic levels. After the sequences were quality-filtered and clustered into OTUs, a network was constructed that links each OTU to the respective sample (Fig. 3). Centroid values were calculated for each node in order to identify OTUs with high centrality. The calculated values ranged from − 56 to + 7 (average − 34.48) for the analyzed dataset. OTUs with the highest relative abundances (OTU52, OTU58, OTU27, OTU11, OTU72, and OTU71) were also shown to be within a centroid distance that was closer than the average distance for all identified enterobacterial OTUs (Fig. 3). This indicates a high centrality of these OTUs in the network. Across the dataset, 52 distinct de novo enterobacterial OTUs were assigned after removal of chimeric sequences and non enterobacterial hits from the BIOM file. Furthermore, 14 OTUs were mapped to the enterobacterial core microbiome at 100% samples size (OTU present in every sample across phyllosphere and rhizosphere). At a 50% fraction of all samples (Additional file 1: Figure S2), 37 OTUs (around 71% of all detected OTUs) were considered as core, taking the nature of the habitat into account. All highly abundant OTUs (OTU52, OTU58, OTU27, OTU11, OTU72, and OTU71) were almost exclusively mapped in the core. For robust assignments, taxonomic assignments were supported by neighbor joining trees (Additional file 1: Figure S3–S5) and revealed for OTU52 = *Erwinia*, OTU58 = *Shigella*/*Salmonella*, OTU27 = *Shigella/Escherichia*, OTU11 = *Pantoea*, OTU72 = *Erwinia*, OTU71 = *Rahnella*. To compute alpha and beta diversity indices, each sample was rarified to 3950 reads. Habitat distances based on the two-sided Student’s two-sample *t* test revealed a close grouping for the rhizosphere and a higher heterogeneity for the phyllosphere. Distances between all and within all habitats were similar although with higher scattering between habitats.

### Antibiotic resistances in the arugula metagenome

**Fig. 4:**
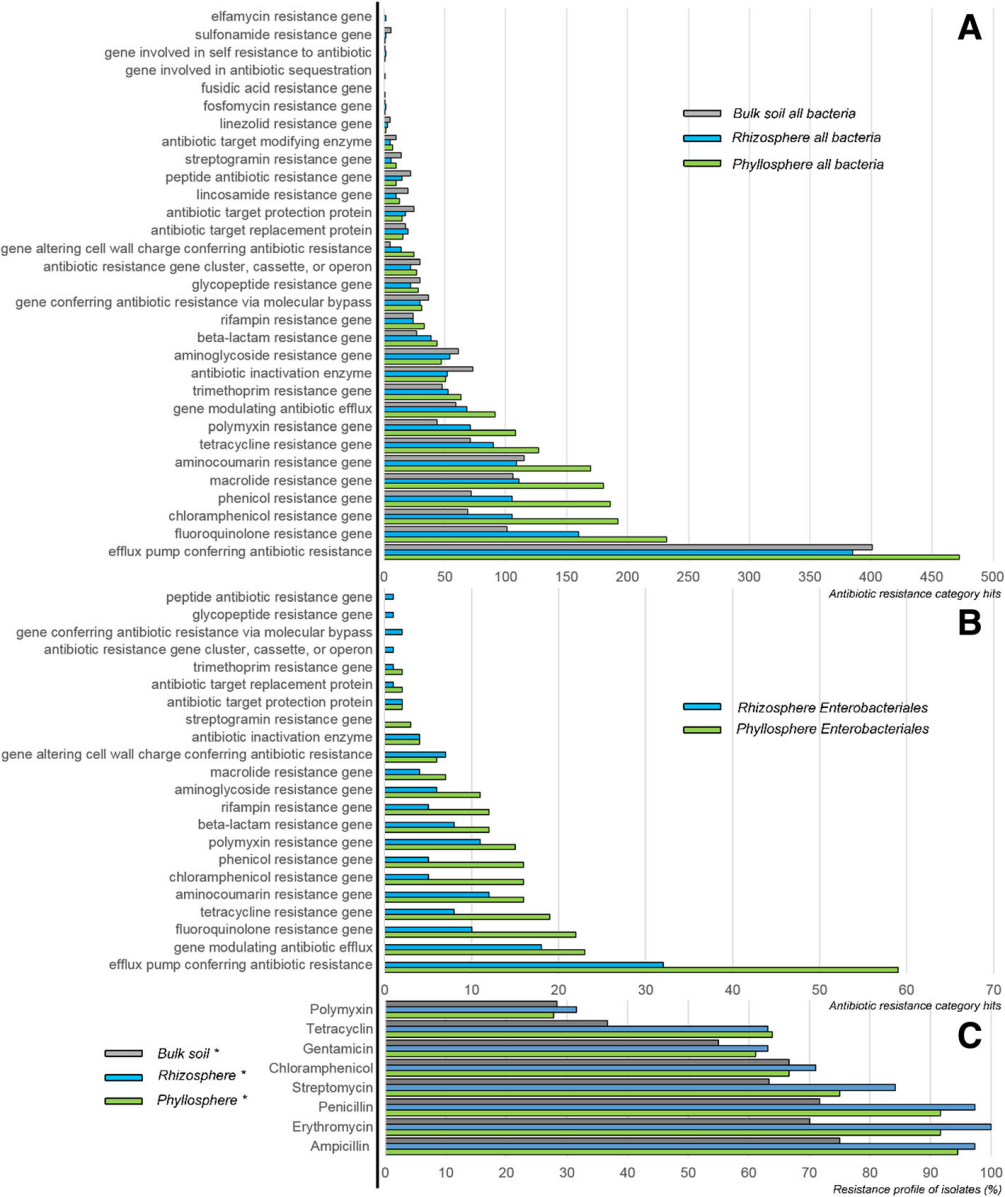
The arugula resistome assessed with metagenome mining and resistance analyses of a strain collection. The CARD-based [45] analysis targets known genes conferring antibiotic resistance. **a** The general analysis of antibiotic resistance in bacteria is based on blastx assignments for 89,462 subsampled contigs from each sample and **b** 5494 respective query contigs for the *Enterobacteriales* fraction. **c** A total of 180 cultivable isolates obtained from arugula plants was subjected to antibiotic susceptibility testing

**Fig. 5:**
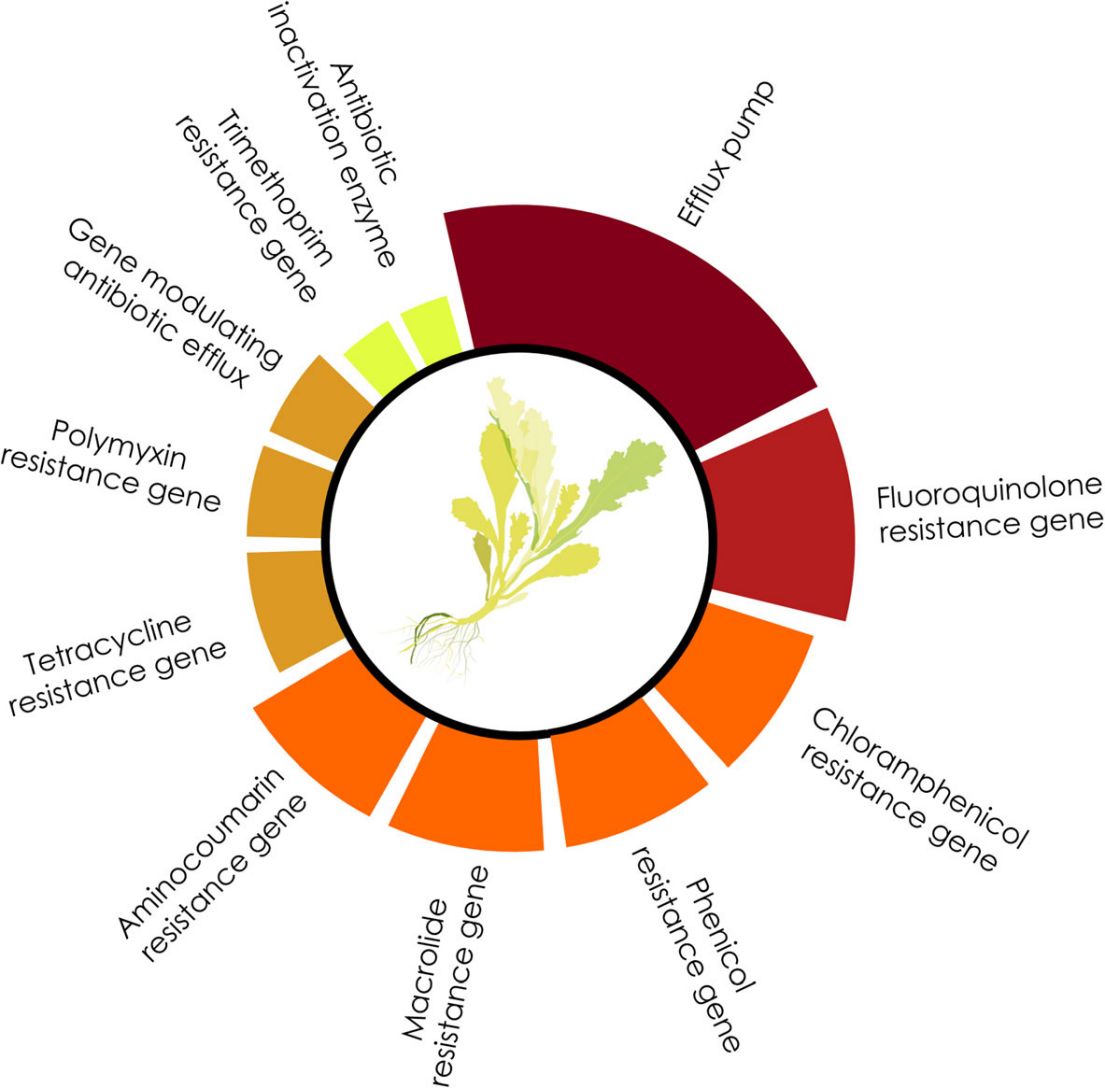
Schematic profile of predominant antibiotic resistances of bacteria in edible plant parts. The eleven most abundant resistance mechanisms identified within the CARD-based [45] analysis are displayed in the outer, discontinuous circle. Areas of the segments correlate with the respective number of contigs that were assigned to distinct CARD categories

To determine the occurrence of antibiotic resistances in the metagenomes, BLAST searches against the Comprehensive Antibiotic Resistance Database (CARD; [45]) sequence collection were carried out. Assembled metagenomes were separately assessed on bacteria and *Enterobacteriales* taxonomic ranks (Fig. 4a, b), respectively. Results showed that efflux pumps conferring antibiotic resistance (21.6–26.8% of all hits) were prevalent for all bacteria. This was followed by the more specific resistance gene against fluoroquinolone (6.7–10.6%), chloramphenicol (4.6–8.8%), macrolide (6.9–8.2%). In addition, a broad spectrum covering diverse genes conferring antibiotic resistance was found (Fig. 4a). The phyllosphere metagenome included phenicol resistance (186), aminocoumarine resistance (170), tetracycline resistance (127), polymyxin resistance (108), genes modulating antibiotic efflux (91), trimetroprim resistance (63), antibiotic inactivation enzymes (51), and aminoglycoside resistance (47). A schematic overview of antibiotic resistances that were found in edible plant parts is shown in Fig. 5. Based on 89,462 query contigs for each metagenome, the highest abundance of antibiotic resistance genes was retrieved from the phyllosphere (2185). The arugula rhizosphere (1599) and bulk soil (1495) each had a lower number of genes included in the CARD collection. Most of the included resistance categories found in all bacteria could also be retrieved for *Enterobacteriales* (Fig. 4b), where various antibiotic resistance categories were prevalently associated to the phyllosphere (247 hits), while fewer categories were found for the rhizosphere (144 hits).

### Antibiotic resistance analysis with the Kirby Bauer testing system

A strain library with 180 isolates was constructed following the isolation approach with various cultivation media and samples from the two plant habitats. This strain library was subjected to the standardized testing system (Kirby Bauer; [4]). A high antibiotic resistance profile was observed against most tested antibiotics, which comprised Ampicillin, Chloramphenicol, Erythromycin, Gentamicin, Penicillin G, Polymyxin B, Streptomycin, and Tetracycline. Isolates derived from the rhizosphere and phyllosphere showed higher antibiotic resistance per strain when compared to the soil isolates. More than 50% of the strain library subjected to the antibiotics showed resistance at the tested concentrations. Polymyxin was the only antibiotic with resistance abundance lower than 50%. Eleven strains showed multi resistance toward all subjected antibiotics (Additional file 1: Table S4). These strains were further sequenced and BLAST-searched against the 16S rRNA and nt databases of NCBI. Eight strains belong to the group of *Gammaproteobacteria* (including three *Enterobacteriaceae* members), while two were assigned to *Firmicutes* and one to *Bacteroidetes*. The genera *Pseudomonas* (five isolates) and *Erwinina* (two isolates) were repeatedly identified, while the other resistant isolates belonged to different bacterial lineages. When the isolates were compared at species level (Additional file 1: Table S4), no overlaps in taxonomic assignments between the three isolate sources were observed.

## Discussion

Our integrative profiling of the microbiota in arugula provided evidence for a specific microbiome composition with an enrichment of members of the *Enterobacteriaceae* especially in the edible plant parts. Their representatives, e.g., *Pantoea*, *Erwinia*, *Pectobacterium*, *Serratia*, and *Yersinia*, are well-equipped with various antibiotic resistances and thus contribute to the overall resistome. These findings confirm our initial hypotheses related to the indigenous arugula microbiome. Unexpectedly, the prevalence of specific resistance mechanisms targeting therapeutic antibiotics in plant-associated microorganisms was only surpassed by the number of identified genes coding for efflux pumps. High frequencies for fluoroquinolone, chloramphenicol, phenicol, macrolide, and aminocoumarin resistances were detected.

### Naturally occurring *Enterobacteriaceae* members dominate arugula’s phyllosphere

The predominance of enterics is in good agreement with previous findings targeting the lettuce (*Lactuca sativa* L.) microbiome [12, 26, 27, 60]. The *Enterobacteriaceae* are a large family of Gram-negative bacteria that are excellent degraders of organic compounds. This family includes many harmless symbionts, serious pathogens, as well as those of ambivalent character, e.g., *Pantoea* and *Erwinia* ([38] [23, 56]), which were among the predominant lineages in the phyllosphere metagenome, as well as in the amplicon datasets dominant in arugula. They were also part of the enterobacterial core-biome and thus likely contribute to the host’s natural biota. Interpretation of the network analyses suggests that *Enterobacteriaceae* with a high relative abundance are not the only opportunistic niche occupiers on the plants. According to our data, it is likely that the soil provides the major reservoir from which enterics can establish and spread within the whole plant. It has already been shown that the leaf age, plant lesions, and nitrogen content affect the bacterial communities and promote the proliferation of enterics [13, 14, 27]. We assume that the arugula phyllobiome favors bacteria that can cope well with the rapidly expanding habitat of the growing leaves. Similar to lettuce or spinach, which also contains high amounts of enterobacteria, leaves of arugula have a similar structure. This provides a different habitat compared to the phyllosphere of other plants, which endorse structurally different microbiota [24]. One aspect that might contribute to the survival of the enterobacterial community could be the tolerance against a broad range of plant-specific metabolites [59] that may be involved in the regulation of the microbial community. The presence of bioactive compounds may shrink the possibilities of other microorganisms to rapidly establish in affected habitats.

### Antibiotic resistances are an additional risk factor associated with opportunistic pathogens

Our data showed an accumulation of *Enterobacteriaceae* in the phyllosphere of arugula with approximately threefold higher abundances, when compared to the rhizosphere. Other plants where high proportions of enterobacteria were found include spinach and bean sprouts sold in grocery stores [41]. Thus, the question arises if a high occurrence of antibiotic resistances could be an additional risk factor for immunocompromised consumers, when coupled with distinct pathogenicity mechanisms. These protective mechanisms naturally occur in microorganism and are even strengthened by various human activities aimed at preventing and treating diseases [44]. An extensive presence of antibiotic resistances was repeatedly shown for various food production systems—mostly due to the preventive use of antibiotics in animal mass production [1]. Our study complements these findings by targeting AR in a leafy green from non-intensive farming, i.e., home gardening. We could show that a broad spectrum of antibiotic resistances was present in the indigenous microbiota and that resistances targeting therapeutic antibiotics unexpectedly prevailed. Arugula is a representative model plant for vegetables that are primarily consumed uncooked in Western cuisine, thus potential pathogens cannot be deactivated by heating. A holistic assessment of the plant resistome showed that the phyllosphere, which constitutes an interface to human health, accounts for most of the identified AR genes. Antibiotic resistance mechanisms were shown to be highly diverse within the microbiota. Similar AR profiles are also found in intensive farming [48]. Remarkably, the most frequent resistances in the arugula microbiome have a significant overlap with the World Health Organization’s list of the most critical antibiotic resistances [20]. While the sole presence of genes related to antibiotic resistances is not evidential for their expression, cultivation-dependent studies provided good means to confirm such findings. In the present study, the high occurrence of resistance-conferring genes in the metagenome was reflected by the tolerance observed in a broad screening of plant-associated isolates. Moreover, members of the *Enterobacteriales* order were shown to contribute to the resistome profile found in the microbiome. We hypothesize that the bioactive metabolites produced by the host plant facilitate the prevalence of AR in the indigenous arugula microbiome. Co-selections were previously shown for metal and antibiotic resistances [3]. Cross-resistances, where the same genetic determined confers resistance to different substances, play an important role in these mechanisms. In the well-studied enterobacterium *Escherichia coli*, exposure to salicylate induces multiple antibiotic resistance by activation of the corresponding *mar* operon [19]. It has been already highlighted that plant-associated bacteria and human opportunists are often equipped with the same genetic basis that facilitates manifestation of disease [8]. Arugula-associated enterobacteria reflect this observation particularly well. The results are based on two plant development stages and include plants from a single sampling site; therefore, they provide a first insight into this highly interesting microenvironment. In order to obtain deeper insights related to the formation, dynamics, and localization (plasmid or genomic) of the antibiotic resistances, additional studies of larger sample sets are required.

## Conclusions

The conducted study allowed a holistic assessment of the enterobacterial population in the *Eruca sativa* Mill. microbiome by combining different cultivation-dependent and cultivation-independent analyses. The occurrence of various *Enterobacteriaceae* members in the core microbiome indicated that they are well adapted to this particular host plant and part of the natural biota. Moreover, their enrichment in above-ground plant parts is an indicator for their competitiveness in a restrictive environment, which is aggravated by the plant’s bioactive metabolites. Metagenomic analyses in combination with isolates screenings provided evidence for the occurrence of various antibiotic resistance mechanisms in the microbiome. These defense mechanisms were mainly against therapeutic antibiotics. So far, such an AR spectrum was not observed in a natural habitat. Therefore, the arugula system provides a unique model to study AR in an ecological context. The implications of the resistance carriers found in this study for human health as well as the interactions of such microorganisms with the surrounding environment and intruding pathogens remain to be elucidated.

## Additional file

Additional file 1: **Table S1.** Sample and processing details. Composite samples were obtained from multiple plants that were homogenized prior to DNA extractions and isolation of bacteria. **Table S2.** Alignment of quality-filtered reads to genomes of the *Brassicacea* plant family. **Table S3.** Assembly statistics for the Velvet-based de novo assembly. For the m*edian coverage*, *N50*, and *Maximum contig length* columns two values are shown. The first value for median coverage represents the number of k-mers, while the second value represents the number of utilized bases. **Table S4.** Multi-resistant isolates identified in antibiotic susceptibility experiments. Colors indicate the isolation source of the isolates. Soil (gray); Rhizosphere (blue); Phyllosphere (green). **Figure S1.** Metadata profile starting 14 days before sampling of plants for the metagenome sequencing. **Figure S2.** Core OTUs assigned to enterobacteria across 50% - 100% of the samples. **Figure S3.** Neighbor joining tree with max. Sequence identity 0.1 (90% similarity) and RefSeq BLAST assignments. OTU 52 is highlighted in yellow. **Figure S4.** Neighbor joining tree with max. Sequence identity 0.1 (90% similarity) and RefSeq BLAST assignments. OTU 58 is highlighted in yellow. **Figure S5.** Neighbor joining tree with max. Sequence identity 0.1 (90% similarity) and RefSeq BLAST assignments. OTU27 is highlighted in yellow. (DOCX 2866 kb)
