# Supplementary material for: Enterobacteriaceae dominate the core microbiome and contribute to the resistome of arugula (Eruca sativa Mill.)
Source: Microbiome. 2019 Jan 29;7:13. doi: 10.1186/s40168-019-0624-7 (PMC6352427; doi:10.1186/s40168-019-0624-7)
Supplement: Supplementary file 1 — Table S1. Sample and processing details. Composite samples were obtained from multiple plants that were homogenized prior to DNA extractions and isolation of bacteria. Table S2. Alignment of quality-filtered reads to genomes of the Brassicacea plant family. Table S3. Assembly statistics for the Velvet-based de novo assembly. For the median coverage, N50, and Maximum contig length columns two values are shown. The first value for median coverage represents the number of k-mers, while the second value represents the number of utilized bases. Table S4. Multi-resistant isolates identified in antibiotic susceptibility experiments. Colors indicate the isolation source of the isolates. Soil (gray); Rhizosphere (blue); Phyllosphere (green). Figure S1. Metadata profile starting 14 days before sampling of plants for the metagenome sequencing. Figure S2. Core OTUs assigned to enterobacteria across 50% - 100% of the samples. Figure S3. Neighbor joining tree with max. Sequence identity 0.1 (90% similarity) and RefSeq BLAST assignments. OTU 52 is highlighted in yellow. Figure S4. Neighbor joining tree with max. Sequence identity 0.1 (90% similarity) and RefSeq BLAST assignments. OTU 58 is highlighted in yellow. Figure S5. Neighbor joining tree with max. Sequence identity 0.1 (90% similarity) and RefSeq BLAST assignments. OTU27 is highlighted in yellow. (DOCX 2866 kb) [file 40168_2019_624_MOESM1_ESM.docx]

# Supplementary material

# *Enterobacteriaceae* dominate the core microbiome and contribute to the resistome of arugula (*Eruca sativa* Mill.)

**Tomislav Cernava^1*^, Armin Erlacher^1,3*^, Jung Soh^2^, Christoph W. Sensen^2,4^, Martin Grube^3^, Gabriele Berg^1^**

^1^ Institute of Environmental Biotechnology, Graz University of Technology, Petersgasse 12, 8010 Graz, Austria

^2^ Institute of Computational Biotechnology, Graz University of Technology, Petersgasse 14, 8010 Graz, Austria

^3^ Institute of Plant Sciences, University of Graz, Holteigasse 6, 8010 Graz, Austria

^4^ BioTechMed Graz, Mozartgasse 12/II, 8010 Graz, Austria

* These authors contributed equally.

**Email addresses:** tomislav.cernava@tugraz.at; armin.erlacher@tugraz.at; jung.soh@tugraz.at; csensen@tugraz.at; martin.grube@uni-graz.at; gabriele.berg@uni-graz.at

**Author for correspondence:**

Gabriele Berg, Graz University of Technology, Institute of Environmental Biotechnology, Petersgasse 12, A-8010 Graz, email: [gabriele.berg@tugraz.at](mailto:gabriele.berg@tugraz.at), telephone: +43 316 873 8310

**Submitted to *Microbiome***

**Running title:** Antibiotic resistances in the *E. sativa* microbiome

**Key words:** antibiotic resistances; microbiome; *Enterobacteriaceae*; raw-eaten vegetables; microbial communities

**Table S1**. Sample and processing details. Composite samples were obtained from multiple plants that were homogenized prior to DNA extractions and isolation of bacteria.

| **Sample type** | **Sampling period** | **Plant growth stage** | **Samples processed for tcDNA extraction** | **NGS method** | **Isolation media** |
| --- | --- | --- | --- | --- | --- |
|  |  |  |  |  |  |
| *Eruca* phyllosphere (all aboveground plant parts) | July | Leaves fully developed | 3 composite samples | Roche 454 pyrosequencing | - |
|  | November | Flowering stage | 1 composite sample | Illumina HiSeq2000 | PDA, MacConkey, KingsB, R2A, MIS, NAII |
| *Eruca* rhizosphere | July | Leaves fully developed | 3 composite samples | Roche 454 pyrosequencing | - |
|  | November | Flowering stage | 1 composite sample | Illumina HiSeq2000 | PDA, MacConkey, KingsB, R2A, MIS, NAII |
| Bulk soil | November | - | 1 composite sample | Illumina HiSeq2000 | PDA, MacConkey, KingsB, R2A, MIS, NAII |

**Table S2.** Alignment of quality-filtered reads to genomes of the *Brassicacea* plant family.

| Sample | Quality-filtered reads | Aligned to *Brassica oleracea* genome | Aligned to *Brassica rapa* genome | Aligned to *Raphanus sativus* genome | Aligned to at least one genome | Not aligned to any genome |
| --- | --- | --- | --- | --- | --- | --- |
| Phyllosphere | 41,867,724 | 10,138,094 (24.21%) | 8,734,400 (20.86%) | 8,873,451 (21.19%) | 12,040,211 (28.76%) | 29,827,513 (71.24%) |
| Rhizosphere | 35,463,395 | 480,897 (1.36%) | 411,291 (1.16%) | 414,820 (1.17%) | 584,141 (1.65%) | 34,879,254 (98.35%) |
| Soil | 27,085,866 | 29,385 (0.11%) | 25,714 (0.09%) | 24,779 (0.09%) | 42,586 (0.16%) | 27,043,280 (99.84%) |

**Table S3**. Assembly statistics for the Velvet-based *de novo* assembly. For the m*edian coverage*, *N50*, and *Maximum contig length* columns two values are shown. The first value for median coverage represents the number of k-mers, while the second value represents the number of utilized bases.

| Sample | *k-mer* | Median coverage | | Number of contigs | N50 | | Maximum contig length | | Total assembly length | Number of reads used |
| --- | --- | --- | --- | --- | --- | --- | --- | --- | --- | --- |
| Phyllosphere | 79 | 2.793893 | 5.863240 | 1,387,116 | 403 | 481 | 152,920 | 152,998 | 306,637,366 | 23,643,355 |
| Rhizosphere | 79 | 1.791781 | 3.760216 | 387,201 | 225 | 303 | 16,916 | 16,994 | 91,405,824 | 3,056,914 |
| Soil | 75 | 1.451613 | 2.883871 | 273,109 | 192 | 266 | 3,562 | 3,636 | 53,493,324 | 1,242,904 |

**Table S4.** Multi-resistant isolates identified in antibiotic susceptibility experiments. Colors indicate the isolation source of the isolates. Soil (grey); Rhizosphere (blue); Phyllosphere (green)

| **Isolate** | **NCBI 16S rRNA database BLAST** | **NCBI nt collection BLAST** |
| --- | --- | --- |
| 35 EAS-0 MacK | *Serratia marcescens* | *Serratia marcescens* |
| 47 EAR-1 MacK | *Pseudomonas kilonensis/corrugata* | *Pseudomonas brassicacearum* |
| 49 EAR-2 MacK | *Pseudomonas brassicacearum* | *Pseudomonas brassicacearum* |
| 51 EAR-2 MacK | *Pseudomonas mendelii* | *Pseudomonas mandelii/syringae* |
| 60 EAP-2 MacK | *Erwinia persicina* | *Erwinia rhapontici* |
| 71 EAS-2 R2A | *Erwinia persicina* | *Erwinia rhapontici* |
| 77 EAR-2 R2A | *Chryseobacterium lactis* | *Chryseobacterium joostei* |
| 135 EAS-2 NA | *Pseudomonas migulae/arsenicoxydans* | *Pseudomonas migulae/brassicacearum* |
| 136 EAR-3 NA | *Bacillus toyonensis/thurigensis* | *Bacillus oryzaecorticis/thuringensis* |
| 150 EAP-3 NA | *Sphingobacterium faecium* | *Sphingobacterium kitahiroshimense* |
| 154 EAP-2 NA | *Pseudomonas helmaticensis* | *Pseudomonas baetica* |


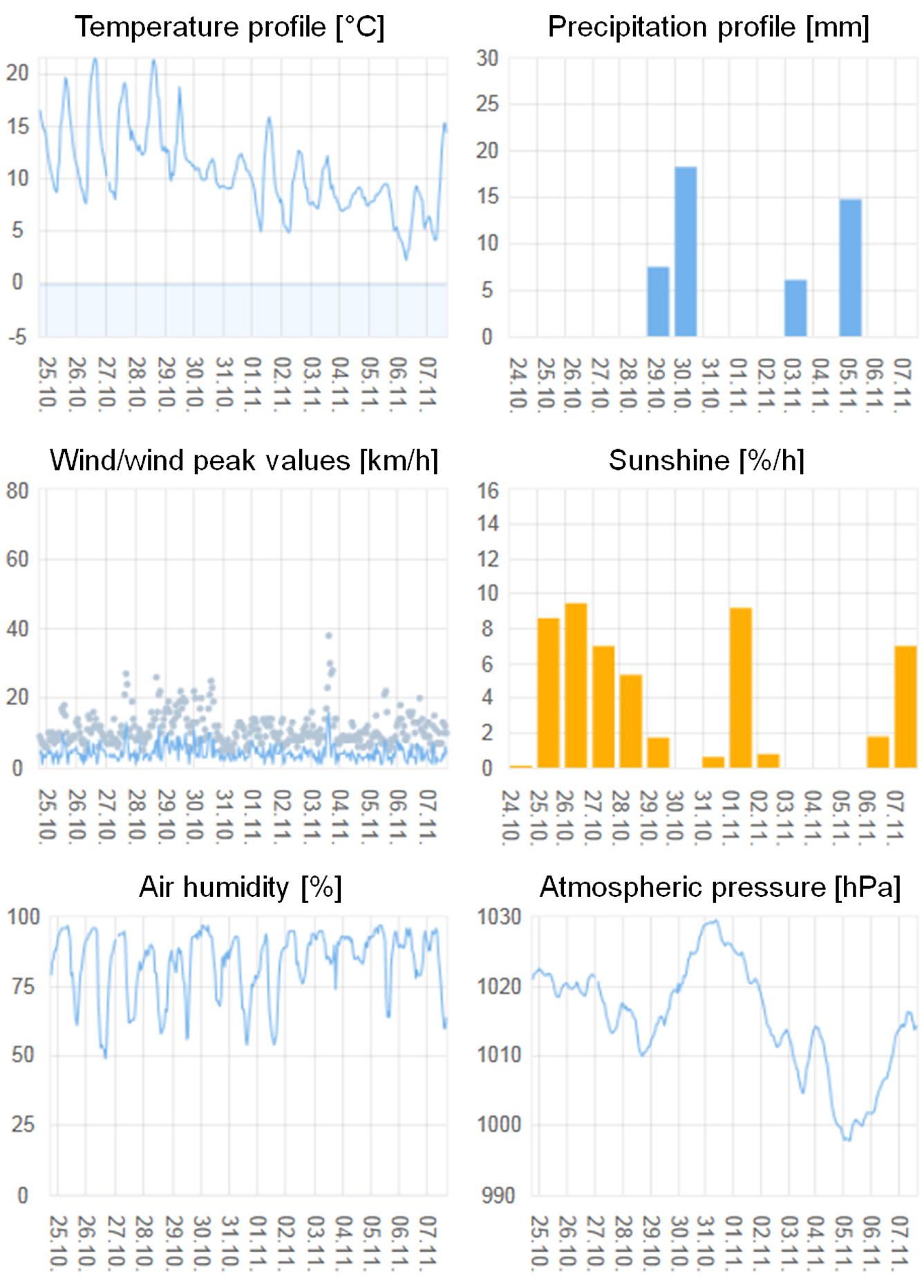


**Figure S1**. Metadata profile starting 14 days before sampling of plants for the metagenome sequencing.


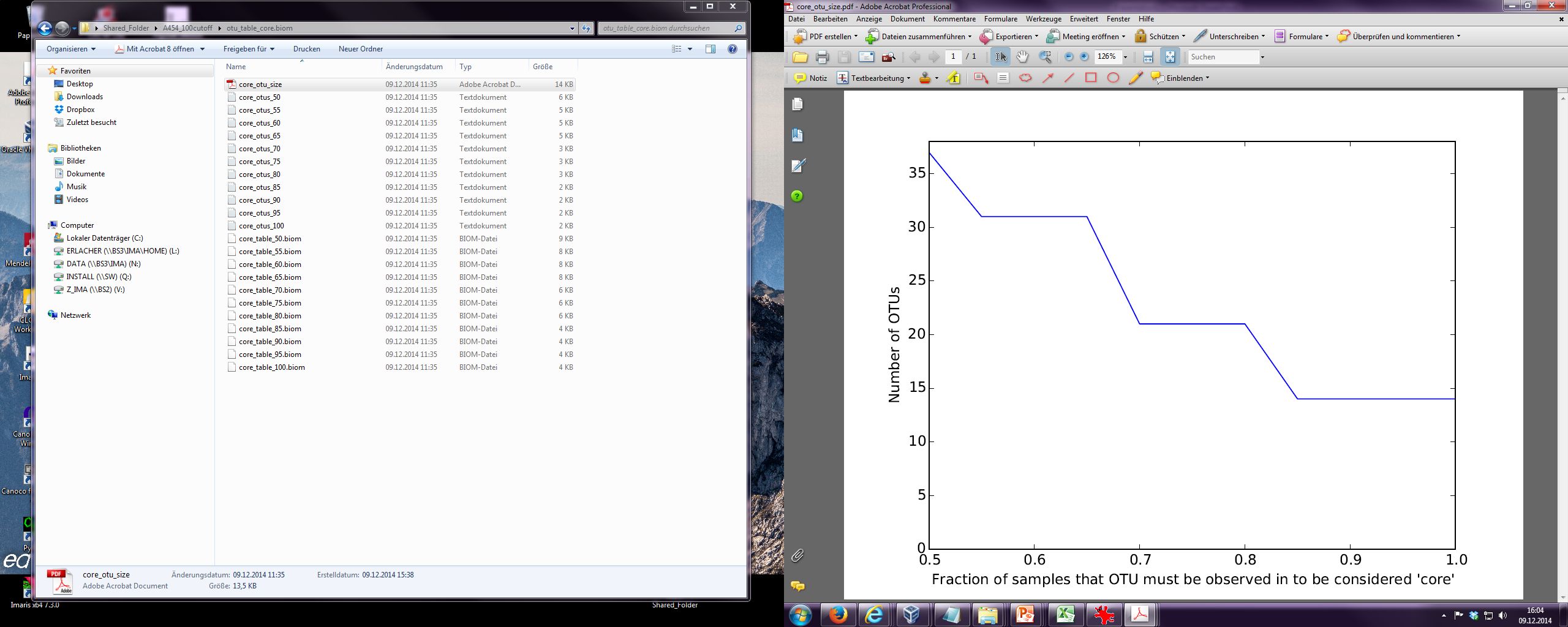


**Figure S2**. Core OTUs assigned to enterobacteria across 50% - 100% of the samples.


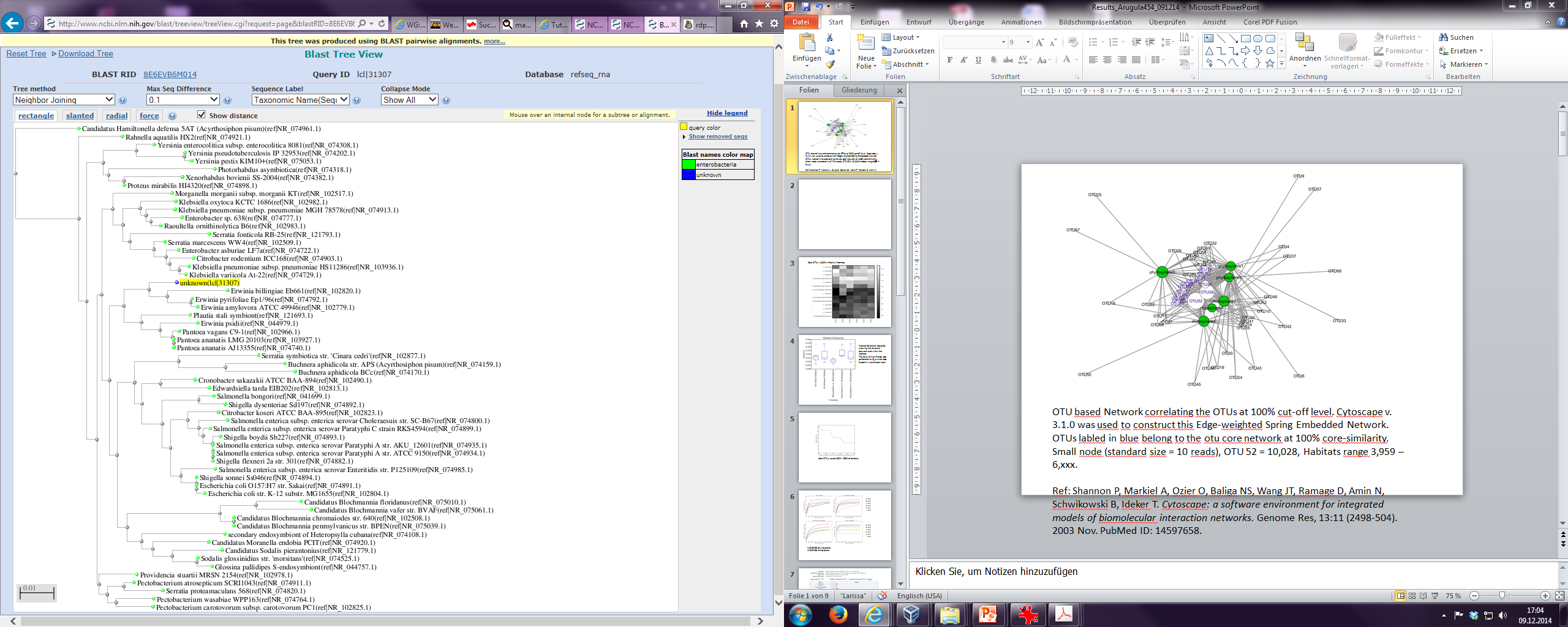


**Figure S3.** Neighbor joining tree with max. sequence identity 0.1 (90% similarity) and RefSeq BLAST assignments. OTU 52 is highlighted in yellow.


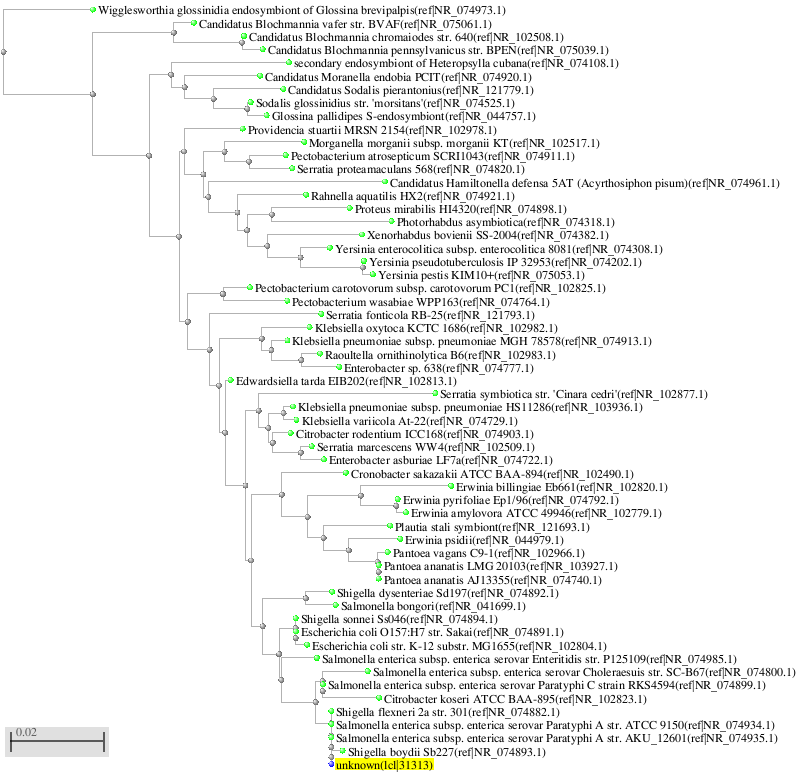


**Figure S4**. Neighbor joining tree with max. sequence identity 0.1 (90% similarity) and RefSeq BLAST assignments. OTU 58 is highlighted in yellow.


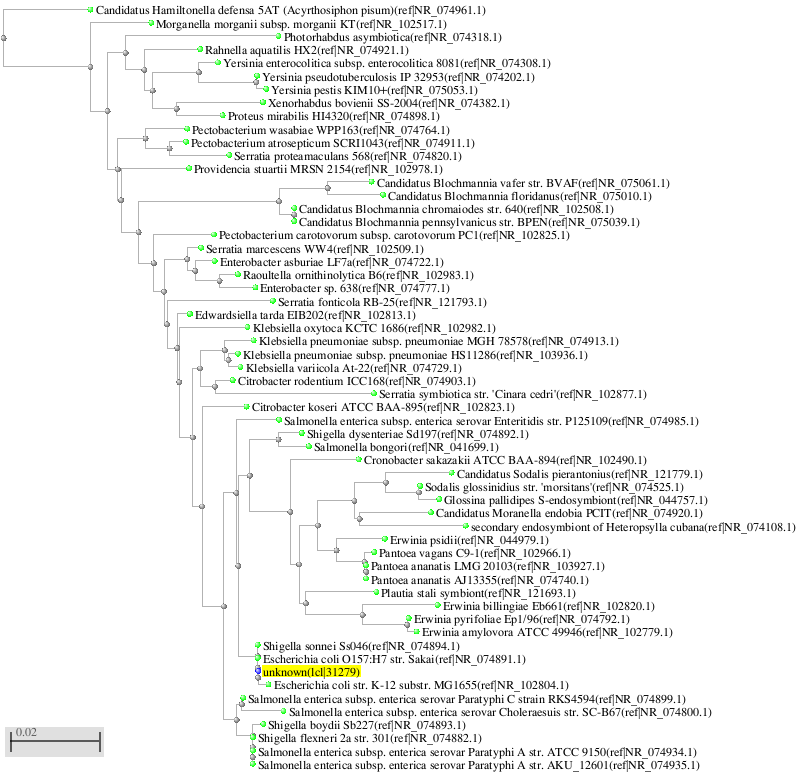


**Figure S5**. Neighbor joining tree with max. sequence identity 0.1 (90% similarity) and RefSeq BLAST assignments. OTU27 is highlighted in yellow.
